# Yield and nutritional quality of soil‐cultivated crisphead lettuce (*Lactuca sativa* L. var. *capitata*) and corn salad (*Valerianella* spp.) harvested at different growing periods

**DOI:** 10.1002/fsn3.3205

**Published:** 2023-01-06

**Authors:** Lovro Sinkovič, Doris Kokalj Sinkovič, Kristina Ugrinović

**Affiliations:** ^1^ Crop Science Department Agricultural Institute of Slovenia Ljubljana Slovenia

**Keywords:** elements, growing period, isovaleric acid, leaf position, salad crops, yield

## Abstract

Crisphead lettuce and corn salad varieties were grown during different growing seasons in the greenhouse or in the field under intensive tillage. They were evaluated for agronomic and nutritional quality to determine the influence of growing period, variety, and in the case of lettuce, leaf position (inner, middle, and outer leaf) on total and marketable yield, color parameters, relative chlorophyll, total sugars, isovaleric acid, total phenolic content (TPC), and multielement composition. Significant differences were found among total yield, color parameters (L*, b*, c*, and *hue*), and relative chlorophyll for crisphead lettuce, and color parameters (L*, a*, b*, c*, and *hue*) and relative chlorophyll for corn salad varieties. Relative chlorophyll was strongly correlated with most color parameters. The growing period affected yield, relative chlorophyll, TPC, total sugars, isovaleric acid, and multielement composition for corn salad, and the color parameter a* and relative chlorophyll for crisphead lettuce. The position of lettuce leaves had a significant effect on TPC, total sugars, and multielement composition. In addition, this study confirmed that farmers and consumers should pay attention to nutritional quality when selecting different lettuce and corn salad varieties.

## Graphical abstract

Crisphead lettuce and corn salad varieties were produced in a greenhouse or open field during different growing periods under intensive soil farming. They were analyzed for several agronomic traits and nutritional quality parameters.

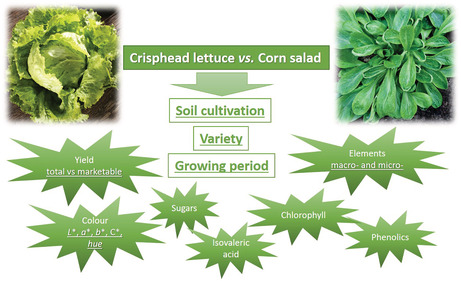

## 1 INTRODUCTION

Market sales of fresh leafy vegetables have changed in recent decades as a result of modern living and changes in consumers' attitudes and lifestyles (Sucheta et al., 2019). Lettuce and corn salad, also known as lamb's lettuce or field salad, are among the most economically important salad crop species and their consumption has spread tremendously due to their high nutritional benefits and as a source of phytochemicals (Noumedem et al., 2017; Pinotti et al., 2020; Yang et al., 2018). Both species are generally consumed raw in salads as a main course or side dish. Therefore, they can contribute significantly to the nutritional content of diets since more nutrients are retained compared to other cooked or processed vegetables (Kim et al., 2016). Dietary patterns that include salads, particularly green leafy vegetables, have been associated with a lower risk of several diseases such as metabolic syndrome, type II diabetes, and cardiovascular diseases (Hoy et al., 2019).

Modern lettuce (*Lactuca sativa* L.) varieties are usually classified into six main types based on morphological similarities, namely crisphead, butterhead, romaine, cos, stem or stalk, and Latin lettuce (Křístková et al., 2008; Mou, 2008; Wei et al., 2021). Among these, crisphead lettuce (var. *capitata* L. *nidus* jäggeri Helm), which includes Batavia, Iceberg, and Eissalat, is one of the most popular types in Europe, the USA, and Brazil (Kim et al., 2016; Noumedem et al., 2017; Schvambach et al., 2020). Crisphead lettuce is traditionally soil cultivated and sold as whole‐head salad that forms a dense head/rosette with leaves branching from a single stalk, although the supply of hydroponically produced ready‐to‐eat baby leaf lettuce is increasing (Pinotti et al., 2020). Corn salad (*Valerianella locusta* L. and *Valerianella eriocarpa* Desv.) is a small annual dicotyledonous plant with a short growth cycle that is consumed mainly for its pleasant sweet taste and soft texture (García‐Valcárcel et al., 2016; Hernández et al., 2021). The plant forms six to seven pairs of loose opposing leaves, which are usually harvested and consumed as a whole rosette (Ceglie et al., 2018).

The agronomic traits and nutritional quality of lettuce and corn salad depend mainly on the species type, variety, and cultivation technique, as both generally have a very short postharvest life and may be stored for up to 15 days at a low temperature near 0°C and relative humidity above 95% (Kotsiras et al., 2016). In addition to varietal differences, the nutritional quality of lettuce can also be influenced by environmental factors such as light, temperature, growing period, agricultural practices, fertilizer use, and storage conditions (Mou, 2012). The development and yield of corn salad plants are strongly influenced by mineral nutrition and environmental stresses (Ceglie et al., 2018). Yield, especially marketable yield, of both salad crop species, is the most important agronomic parameter for growers, which is highly dependent on climatic factors such as temperature, precipitation, daily solar radiation, and relative humidity (Sahin et al., 2016). In Europe, where Spain and Italy are the largest producers, the production of lettuce (including chicory) reached almost 3.9 million tons in 2019 with an average yield of 28.6 t ha^−1^ and 22.5 t ha^−1^, respectively, according to FAOSTAT. According to national data, lettuce was grown on 957 ha and corn salad on 140 ha in Slovenia in 2020. The 10‐year average yield was highly dependent on growing conditions and cultivation technology, reaching 13.3–23.0 t ha^−1^ for lettuce and 4.9–8.2 t ha^−1^ for corn salad.

Lettuce and corn salad are traditionally grown outdoors in family gardens and by specialized vegetable producers in local farms for fresh market production (Šuštar‐Vozlič et al., 2021). Their cultivation and consumption have a long tradition in Slovenia, as evidenced by many varieties registered in the National List of Varieties. In general, the best condition for lettuce growth is cooler temperature conditions in the range 7°C–24°C. Growing lettuce above this range reduces quality and yield and usually results in economically significant physiological disorders such as tip burn, rib discoloration, premature bolting, and russet spotting (Jenni et al., 2013; Masarirambi et al., 2018). Soil cultivation of corn salad mostly takes place in the colder season (autumn–winter–spring period), as it does not require high temperatures for growth (5°C–10°C) and can withstand temperatures as low as −25°C (Žnidarčič, 2016).

From a nutritional standpoint, lettuce and corn salad represent ideal food since they are low in calories, fat, and sodium. Likewise, both species are good sources of important nutrients such as vitamins and minerals, and health‐promoting bioactive compounds such as phenolic acids, flavonoids, and glycosides (Camejo et al., 2020; Kim et al., 2016). For consumers, color is one of the most important sensory parameters in the selection and acceptance of fresh leafy vegetables used for salads (Colonna et al., 2016). Color preference is a subjective parameter, but consumers associate color with freshness (Lee et al., 2013). In addition, color is an important quality parameter that depends on the chemical, biochemical, microbiological, and physical changes that occur during growth, ripening, and postharvest handling (Pathare et al., 2013). Color can be described by different color coordinate systems, of which CIELAB is the most commonly used for food (Markovic et al., 2013). The value L* describes the brightness of the color, a* the position between red and green, and b* the position between yellow and blue, while *hue* means the color tone, and chroma (C*) the color purity (Pathare et al., 2013). Chlorophylls are the major photosynthetic pigments widely distributed in green vegetables and are responsible for the color of green plants. Due to their antioxidant, anti‐inflammatory, and antitumoral effects, various human health benefits have been reported (Kang et al., 2018). Phenolic compounds such as phenolic acids and flavonoids are phytochemicals widely distributed in leafy vegetables and are also considered compounds with antioxidant properties (Negrao et al., 2021; Zhou et al., 2020).

The corn salad belongs to the Valerianaceae (Caprifoliaceae) or valerian family, whose plants often have a strong and specific odor associated with the presence of organic acids and terpenes. Isovaleric acid is a branched‐chain volatile organic acid with five carbon atoms and is mainly responsible for the characteristic aroma of valerian (Lanje et al., 2020). The presence of valeric acids and isovaleric acid has been reported for valerian roots (*Valeriana officinalis*), but there are no reports on their content in corn salad leaves (Shi et al., 2021).

The objectives of this study were as follows: (1) evaluate the yield, color parameters, and relative chlorophyll content (SPAD) of several crisphead lettuce and corn salad varieties cultivated at different growing periods; (2) assess the variation in total phenolic content, total sugars, and multielemental composition in the leaves of the studied species and varieties cultivated at different growing periods; (3) to study the accumulation of isovaleric acid content in the leaves of corn salad varieties cultivated at different growing periods; and (4) to determine the nutritional quality and differences in crisphead lettuce according to leaf position (outer, middle, and inner).

## 2 MATERIALS AND METHODS

### 2.1 Plant material and experimental design

A total of nine varieties of two salad crop species were used in this study. Four varieties of crisphead lettuce (*L. sativa* L. var. *capitata*): “Leda,” “Posavka,” “Ljubljanska ledenka,” and “Trnovska ledenka”; and five varieties of corn salad, four of the species *V. locusta* L.—“Ljubljanski,” “Žličar,” “Gala,” and “D'Olanda a seme grosso,” and one of the species *Valerianella eriocarpa* Desv.—“Pomladin.” A list of all these varieties together with the origin of the seed and some leaf characteristics are presented in Table 1. Soil cultivation experiments were conducted in spring and autumn of 2017 in a greenhouse and in an open field of the Agricultural Institute of Slovenia in Jablje (46°7´N 14°34´E; altitude 308 m a.s.l.; sub‐Alpine climate) according to standard production practice, which includes regular hoeing, weeding, and irrigation. The soil of the experimental field is deep, heavy, slightly alkaline (pH = 7.4), and overstocked with K_2_O and P_2_O_5_. For the crisphead lettuce varieties, two (I—spring greenhouse, II—spring open field), and for the corn salad varieties, three growing periods (I—spring greenhouse, II—spring open field, III—autumn open field) were conducted and compared. The experiments were set up as random blocks with three replicates. Lettuce seedlings were grown in polystyrene plates (104 cells) filled with peat substrate (Potgrond P, Klasmann) in a heated greenhouse before being planted out, while corn salad was sown directly. The detailed design of the trials and the crop management practices are summarized in Table 2. In 2017, March was warm and sunny, as was the first half of April, while the last third of April was colder, with 2 days of frost and abundant precipitation according to data from the Slovenian Environment Agency. The lettuce trial in the open field was therefore protected with agrotextile. May started with cold weather, from the second decade the days became warmer. Precipitation was lower than usual. Autumn began with a cold, cloudy, and rainy September. In contrast to September, dry weather prevailed in October with only a few rainy days. The first frost occurred in the last days of October.

**TABLE 1:** List of crisphead lettuce (*Lactuca sativa* L. var. *capitata*) and corn salad (*Valerianella locusta* L. and *Valerianella eriocarpa* Desv.) varieties included in the study

Variety name	Variety maintainer (seed supplier in Slovenia^a^)	Leaf: Color	Leaf: Anthocyanins
Crisphead lettuce (*Lactuca sativa* L. var. *capitata*)			
Leda	Semenarna Ljubljana, Slovenia	Light green	Absent
Posavka	Consorzio Sativa Società Cooperativa Agricola, Italy (Planta Prelesje)	Dark green	Present
Ljubljanska ledenka	Agricultural Institute of Slovenia, Slovenia	Very light green	Absent
Trnovska ledenka	Horticulture farm Pustovrh, Slovenia	Light green	Present
Corn salad (*Valerianella locusta* L. and *Valerianella eriocarpa* Desv.)			
Ljubljanski	Semenarna Ljubljana, Slovenia	Medium green	Present
Pomladin	Semenarna Ljubljana, Slovenia	Light green/yellow	Absent
Žličar	Semenarna Ljubljana, Slovenia	Medium green	Absent
Gala	HM Clause, Italy (Cabo)	Dark green	Absent
D'Olanda a seme grosso	Suba Seeds co., Italy (Semina)	Medium green	Absent

^a^ When seed supplier is not the same as seed maintainer.

**TABLE 2:** Cultivation practices for growing crisphead lettuce varieties in two growing periods and corn salad varieties in three growing periods during the 2017 season

Management practice	Crisphead lettuce growing period (in 2017)	
	I—spring (greenhouse)	II—spring (open field)
Location	Jablje, Slovenia	
Plot size	2.7 m^2^; randomized blocks in three repetitions	
Planting distance	30 × 30 cm; three rows on raised beds	
Plant density	6.7 plant m^−2^	
No. of plants/plot	18	
Previous crop	Tomato, eggplant	Celery, carrot
Basic fertilization	Cattle manure (20 t ha^−1^)—before plowing	
Fertilization I.	102 kg ha^−1^ N, 60 kg ha^−1^ P_2_O_5_, 270 kg ha^−1^ K_2_O	28 kg ha^−1^ N, 80 kg ha^−1^ P_2_O_5_, 120 kg ha^−1^ K_2_O
Fertilization II.	19 kg ha^−1^ N, 6 kg ha^−1^ P_2_O_5_, 20 kg ha^−1^ K_2_O, 3 kg ha^−1^ MgO	46 kg ha^−1^ N
Sowing	February 1st	
Transplanting	March 16th	March 21st
Harvest	May 3rd to 8th	May 22nd to 25th

Management practice	Corn salad growing period (in 2017)		
	I—spring (greenhouse)	II—spring (open field)	III—autumn (open field)
Location	Jablje, Slovenia		
Plot size	1.5 m^2^; randomized blocks in three repetitions		
Planting distance	15 × 3 cm; five rows in raised beds		
Plant density	100 plant m^−2^		
Previous crop	Bell pepper	Kale	Lettuce
Basic fertilization	Cattle manure (20 t ha^−1^)—before plowing		
Fertilization I	102 kg ha^−1^ N, 60 kg ha^−1^ P_2_O_5_, 270 kg ha^−1^ K_2_O	28 kg ha^−1^ N, 80 kg ha^−1^ P_2_O_5_, 120 kg ha^−1^ K_2_O	60 kg ha^−1^ N, 60 kg ha^−1^ P_2_O_5_, 240 kg ha^−1^ K_2_O
Fertilization II	28.5 kg ha^−1^ N, 9 kg ha^−1^ P_2_O_5_, 30 kg ha^−1^ K_2_O, 4.5 kg ha^−1^ MgO	46 kg ha^−1^ N	18 kg ha^−1^ N
Sowing	March 10th	March 21st	August 18th
Thinning	April 7th	May 11th	October 24th
Harvest	April 26th to 28th	May 26th to June 1st	November 17th

### 2.2 Yield evaluation

Crisphead lettuce and corn salad plants were harvested at the commercial maturity stage, depending on the variety. For yield evaluation, all plants in each plot were cut at the base and cleaned of damaged and discolored bottom leaves, which were weighted as waste yield, while the remainder represented marketable yield. An electronic field scale (reading to 2 g) was used to weigh the yield. Total yield was calculated as the sum of waste and marketable yield. The results of marketable waste and total yield are expressed as weight in t ha^−1^ and were calculated as the mean of three replicates.

### 2.3 Color parameters and relative chlorophyll content

Leaf color of the crisphead lettuce and corn salad varieties studied was measured on mature plants before harvest using a colorimeter (CR‐400; Minolta) and the CIE parameters (L*, a*, b*) were determined. Fifteen individual measurements per variety were made in each plot (*n* = 45). Hue angle (*hue*) was determined as arctg (b*/a*), and Chroma (C*) was calculated as (a* ^2^ + b* ^2^)^1/2^. The chlorophyll meter SPAD‐502Plus (Minolta) was used to evaluate the relative chlorophyll content. Measurements were made on the fully developed leaves in the center of the lettuce or corn salad plants. Ten individual measurements per variety were taken in each plot, with each measurement comprising 10 readings (*n* = 30).

### 2.4 Nutritional quality parameters

#### 2.4.1 Sample preparation

At harvest, the clean and undamaged outer, middle, and inner leaves of crisphead lettuce were sampled. Leaves from three individual lettuce plants from each plot were collected as bulk samples. For corn salad, approximately 100 g of cleaned and undamaged rosettes from each plot were sampled and the bulk of all three replicates was prepared for each variety. Samples of both species were immediately frozen with liquid nitrogen and lyophilized (Gamma 1–20, Martin Christ). The lyophilized samples were homogenized using a laboratory ball mill (Retsch MM400) before further analysis.

#### 2.4.2 Total sugars determination

Total sugars were analyzed according to the method for the analysis of feed (EC No. 152/2009). In this method, total sugars were extracted in dilute ethanol and the solution was clarified with Carrez solutions I and II. After eliminating the ethanol, the amount of sugar was determined by the Luff–Schoorl method. Results were expressed in g kg^−1^ on a dry weight basis.

#### 2.4.3 (Iso)valeric acid content

Valeric acid and isovaleric acid were extracted from the homogenized samples with Milli‐Q water according to our developed method for the analysis of animal feeds. The water extracts were acidified with sulfuric acid (pH <2) to obtain free acids (R‐COOH), which were extracted with diethyl ether (Merck) and analyzed with a gas chromatograph using a flame ionization detector. The acids were separated on a 10 m × 0.53 mm × 1.00 μm FFAP column using a temperature gradient in splitless mode. The injector was maintained at 220°C and the detector at 300°C, and the nitrogen flow (carrier gas) was 8 ml min^−1^. The injected volume into the column was 1 μl, which was maintained at 80°C for 5 min. Then, the temperature was increased by 5°C min^−1^ up to 130°C, and by 30°C min^−1^ up to 200°C. The final temperature was maintained for 8 min. The retention times of valeric acid and isovaleric acid were 9.4 min and 11.0 min, respectively. The analytical standards for valeric acid (*n*‐valeric acid and pentanoic acid, CAS 109‐52‐4) and isovaleric acid (3‐methylbutanoic acid and 3‐methylbutyric acid, CAS 503‐74‐2) were from Sigma‐Aldrich (USA). All valeric acid was in the form of isovaleric acid. Isovaleric acid content results were expressed in g kg^−1^ on a dry weight basis.

#### 2.4.4 Total phenolic content

Samples used for the determination of total phenolic content (TPC) were extracted with 70% ethanol and centrifuged at 13,200× g for 5 min before analysis. TPC was determined according to the Folin–Ciocalteu method as first described by Singleton and Rossi (1965), with minor modifications. The absorbance was measured after 50 min of incubation at room temperature, using a spectrophotometer (Agilent Technologies Cary 8454 UV–Vis) at 765 nm, against deionized water as a blank. Calibration was performed using an 8‐point standard curve of gallic acid (Fluka). The TPC of the samples was determined in triplicate and expressed in mg g^−1^ gallic acid equivalents (GAE) on a dry weight basis.

#### 2.4.5 Multielemental analyses using ICP‐MS

Homogenized samples (250 mg) mixed with 6.0 ml of 65% HNO_3_ (Suprapur, Merck) and 2 ml of 30% H_2_O_2_ (Suprapur, Merck) were digested in PTFE vessels using a Milestone ETHOS 1600 microwave system. The digested samples were cooled and diluted to 50 ml with Milli‐Q water. Prior to analysis, the digested samples were diluted by a factor of 20 and consisted of 1% HNO_3_. The contents of the elements were determined using an Agilent ICP‐MS 7900 with the Octopole Reaction System (ORS^4^). The sample introduction system consisted of a quartz double‐pass spray chamber and a MicroMist nebulizer. Nickel sampler and skimmer cones were used. Helium (He) was used as the reaction gas at a flow rate of 5.0 ml min^−1^ in He mode and 10.0 ml min^−1^ in HEHe mode. The isotopes monitored were ^24^Mg, ^31^P, ^34^ S, ^39^K, ^43^Ca, ^52^Cr, ^55^Mn, ^56^Fe, ^66^Zn, and ^95^Mo. Most elements were measured in the He mode, while P and S were measured in the HEHe mode. Quantitative determinations of the elements were performed using the external calibration method. The calibration curve was prepared using the IV‐STOCK‐50 standard solution (Inorganic Ventures); P and S individual standard solutions (Inorganic Ventures) were added separately to the mixture. Analytical blanks, independent quality control (QC) standards, and standard reference material were used for quality control. QC standards were prepared from ICP multielement standard solution VIII (Merck, 109,492) and ICP multielement standard solution XVI (Merck, 109,487). A certified reference material (NIST SRM 1573a tomato leaves, Gaithersburg) was used to check the accuracy. All sample results are reported on a dry weight basis and are expressed as macroelements (g kg^−1^) or microelements (mg kg^−1^).

### 2.5 Data analysis

Statistical analysis of yield data was performed using Statgraphics Centurion XVI (Statgraphics Centurion, 2009). Data on nutritional quality parameters were statistically analyzed using R Commander software (version 4.0.3). Descriptive statistics included mean, standard deviation, minimum (min), and maximum (max). For normally distributed data, the statistical significance of differences between varieties and growing periods was determined by analysis of variance (ANOVA). To test which means were significantly different from each other, the least significant difference (LSD) test was used to compare the means of growing periods and, in the case of lettuce, leaf position, whereas Duncan's test was used to compare the means of varieties. Differences at *p* ≤ .05 were considered statistically significant. The Pearson product–moment correlation test was performed to evaluate the correlation between color parameters and relative chlorophyll content.

## 3 RESULTS AND DISCUSSION

### 3.1 Yield and color measures

During the spring growing periods, lettuce and corn lettuce plants developed well and relatively quickly in the greenhouse and outdoors, while the harvest of fall crops was hampered by the cold, cloudy, and rainy September. For lettuce, the growing cycle from transplanting to harvest for the lettuce varieties tested was from 48 to 53 days in the growing period I (spring greenhouse) and 62–65 days in period II (spring open field). In both periods, “Ljubljanska ledenka” and “Leda” varieties were harvested earlier than “Posavka” and “Trnovska ledenka.” The total yield of soil‐grown crisphead lettuce ranged from 36.00 to 58.64 t ha^−1^, while the marketable yield ranged from 24.54 to 43.03 t ha^−1^ (Table 3). Thus, marketable yield accounted for 59–81% of the total yield, depending on the variety and the growing period. When comparing different growing periods, i.e., I (spring greenhouse) vs. II (spring open field), the marketable and total yield of crisphead lettuce in the greenhouse were slightly higher for most varieties and the percentage of waste yield was slightly lower, except for “Leda.” However, these differences were not significant (Table 4). The mean values of marketable yield, total yield, and percentage of waste yield of crisphead lettuce in growing period I were 32.36 t ha^−1^, 44.15 t ha^−1^, and 27%, respectively, and in growing period II were 30.25 t ha^−1^, 43.77 t ha^−1^, and 31%, respectively. On average for both growing periods, the highest marketable yield was obtained for the variety “Posavka” (37 t ha^−1^), followed by “Trnovska ledenka” (34 t ha^−1^), “Leda” (28 t ha^−1^), and “Ljubljanska ledenka” (26 t ha^−1^). In both growing periods, the variety “Posavka” had a significantly higher total yield than “Ljubljanska ledenka” and “Leda.” The same was true for marketable yield in the greenhouse trial, while in the open‐field trial there was no difference between the varieties due to the significantly higher waste yield of the variety “Posavka.”

**TABLE 3:** The influence of growing period on yield, color parameters, and chlorophyll—SPAD values, and correlations for chlorophyll—SPAD with color measures of four crisphead lettuce and five corn salad varieties

Variety	Growing period	Yield (t ha^−1^)		Color parameters^1^					Chlorophyll—SPAD^2^
		Marketable	Total	L*	a*	b*	C*	Hue	
Crisphead lettuce									
Posavka	I	43.03	58.64	44.77	−14.45	24.86	28.77	59.82	31.40
	II	30.20	51.09	45.02	−13.43	24.33	27.82	61.02	34.00
	*Mean*	*36.62*	*54.87*	*44.90*	*−13.94*	*24.60*	*28.30*	*60.42*	*32.70*
Ljubljanska ledenka	I	27.41	37.41	52.30	−13.87	30.27	33.31	65.37	15.83
	II	24.54	36.00	52.50	−11.82	29.15	31.47	67.94	17.80
	*Mean*	*25.98*	*36.71*	*52.40*	*−12.85*	*29.71*	*32.39*	*66.66*	*16.82*
Leda	I	23.54	32.43	51.67	−14.89	31.12	34.51	64.40	18.33
	II	33.15	41.06	49.64	−13.27	29.80	32.65	65.94	23.20
	*Mean*	*28.35*	*36.75*	*50.66*	*−14.08*	*30.46*	*33.58*	*65.17*	*20.77*
Trnovska ledenka	I	35.46	48.10	51.64	−14.65	32.07	35.27	65.45	20.60
	II	33.09	46.93	51.12	−12.90	31.18	33.77	67.49	22.93
	*Mean*	*34.28*	*47.52*	*51.38*	*−13.78*	*31.63*	*34.52*	*66.47*	*21.77*
*Mean I*		*32.36*	*44.15*	*50.10*	*−14.47*	*29.58*	*32.97*	*63.76*	*21.54*
*Mean II*		*30.25*	*43.77*	*49.57*	*−12.86*	*28.62*	*31.43*	*65.60*	*24.48*
Corel with Chl		n.a.	n.a.	−0.97	−0.23	−0.85	−0.81	−0.79	n.a.
Corn salad									
Ljubljanski	I	3.20	3.28	42.51	−15.45	24.82	29.24	58.07	28.70
	II	5.12	5.64	43.08	−13.80	21.70	25.74	57.47	39.30
	III	4.88	5.01	39.82	−13.88	17.66	22.47	51.76	49.33
	*Mean*	*4.40*	*4.64*	*41.80*	*−14.38*	*21.39*	*25.82*	*55.77*	*39.11*
Žličar	I	2.81	2.98	40.50	−14.57	24.26	28.31	58.98	30.53
	II	4.66	5.09	42.51	−13.53	20.24	24.36	56.28	38.97
	III	4.30	4.45	38.96	−13.51	16.93	21.67	51.36	46.73
	*Mean*	*3.92*	*4.17*	*40.66*	*−13.87*	*20.48*	*24.78*	*55.54*	*38.74*
Pomladin	I	3.14	3.36	51.28	−15.46	34.30	37.63	65.73	20.17
	II	4.75	5.42	47.10	−15.22	30.54	34.16	63.46	28.27
	III	3.35	3.50	50.49	−15.93	29.61	33.63	61.73	25.63
	*Mean*	*3.75*	*4.09*	*49.62*	*−15.54*	*31.48*	*35.14*	*63.64*	*24.69*
Gala	I	3.19	3.35	41.30	−14.20	20.50	24.95	55.22	38.47
	II	5.56	6.17	41.88	−12.45	17.03	21.12	53.72	49.10
	III	3.11	3.35	38.96	−12.29	14.15	18.74	48.99	53.17
	*Mean*	*3.95*	*4.29*	*40.71*	*−12.98*	*17.23*	*21.60*	*52.64*	*46.91*
D'Olanda a seme grosso	I	3.63	3.82	43.43	−15.29	24.21	28.64	57.71	31.13
	II	5.64	6.26	42.22	−13.55	20.61	24.68	56.64	43.60
	III	4.88	4.98	39.50	−14.27	18.25	23.17	51.96	48.27
	*Mean*	*4.72*	*5.02*	*41.72*	*−14.37*	*21.02*	*25.50*	*55.44*	*41.00*
*Mean I*		*3.19*	*3.36*	*43.80*	*−14.99*	*25.62*	*29.75*	*59.14*	*29.80*
*Mean II*		*5.15*	*5.72*	*43.36*	*−13.71*	*22.02*	*26.01*	*57.51*	*39.85*
*Mean III*		*4.10*	*4.26*	*41.55*	*−13.98*	*19.32*	*23.94*	*53.16*	*44.63*
Corel with Chl		n.a.	n.a.	−0.81	0.88	−0.95	−0.96	−0.94	n.a.

*Note*: Data are mean (^1^ *n* = 45, ^2^ *n* = 30).

Abbreviations: I, spring (greenhouse); II, spring (open field); III, autumn (open field); C*, chroma; Chl, chlorophyll; Corel, correlation; *hue*, hue angle; n.a., not applicable.

**TABLE 4:** ANOVA significance of four lettuce varieties cultivated in two growing periods (depending on leaf position) and five corn salad varieties cultivated in three growing periods

Variable		Yield		Color parameters					Chlorophyll–SPAD	TPC	Total sugars	Isovaleric acid	Macroelement					Microelement				
		Total	Marketable	L*	a*	b*	C*	Hue					Mg	P	S	K	Ca	Cr	Mn	Fe	Zn	Mo
Crisphead lettuce																						
Variety	Posavka	a	ns	a	ns	a	a	a	B	ns	ns	/	ns	ns	ns	ns	ns	ns	ns	ns	ns	ns
	Ljubljanska ledenka	c	ns	b	ns	b	ab	b	a	ns	ns	/	ns	ns	ns	ns	ns	ns	ns	ns	ns	ns
	Leda	c	ns	b	ns	b	b	ab	a	ns	ns	/	ns	ns	ns	ns	ns	ns	ns	ns	ns	ns
	Trnovska ledenka	b	ns	b	ns	b	b	b	a	ns	ns	/	ns	ns	ns	ns	ns	ns	ns	ns	ns	ns
Growing period	I	ns	ns	ns	a	ns	ns	ns	a	ns	ns	/	ns	ns	ns	ns	ns	ns	ns	ns	ns	ns
	II	ns	ns	ns	b	ns	ns	ns	b	ns	ns	/	ns	ns	ns	ns	ns	ns	ns	ns	ns	ns
Leaf position	Outer	/	/	/	/	/	/	/	/	b	a	/	b	a	b	c	b	b	b	b	ns	b
	Middle	/	/	/	/	/	/	/	/	a	b	/	a	b	a	b	a	a	a	a	ns	a
	Inner	/	/	/	/	/	/	/	/	a	c	/	a	b	a	a	a	a	a	a	ns	a
Corn salad																						
Variety	Ljubljanski	ns	ns	a	ab	a	a	ab	ab	ns	ns	ns	ns	ns	ns	ns	ns	ns	ns	ns	ns	ns
	Žličar	ns	ns	a	ab	a	a	ab	ab	ns	ns	ns	ns	ns	ns	ns	ns	ns	ns	ns	ns	ns
	Pomladin	ns	ns	b	a	b	b	b	a	ns	ns	ns	ns	ns	ns	ns	ns	ns	ns	ns	ns	ns
	Gala	ns	ns	a	b	a	a	a	b	ns	ns	ns	ns	ns	ns	ns	ns	ns	ns	ns	ns	ns
	D'Olanda a seme grosso	ns	ns	a	ab	a	a	ab	ab	ns	ns	ns	ns	ns	ns	ns	ns	ns	ns	ns	ns	ns
Growing period	I	a	a	ns	ns	ns	ns	ns	a	c	a	b	b	ab	b	c	b	a	ns	ns	c	a
	II	b	b	ns	ns	ns	ns	ns	ab	b	b	b	a	a	b	b	b	ab	ns	ns	a	b
	III	b	b	ns	ns	ns	ns	ns	b	a	c	a	ab	b	a	a	a	b	ns	ns	b	b

*Note*: Different letters (a, b, c) in the columns are significantly different (*p* < .05, characteristic differences among varieties, growing periods, and leaf position).

Abbreviations: I, spring (greenhouse); II, spring (open field); III, autumn (open field); C*, chroma; *hue*, hue angle; ns: not significant; TPC, total phenolic content.

Growth cycles (from sowing to harvest) for the corn salad ranged from 47 to 49 days in growth period I (spring greenhouse), from 66 to 72 days in growth period II (spring open field), and 91 days in growth period III (autumn open field). In both spring trials, “Ljubljanski,” “Žličar,” and “D'Olanda a seme grosso” were harvested earlier than “Pomladin” and “Gala,” while in the autumn growing period, all varieties were harvested on the same day. The total yield for corn salad ranged from 2.98 to 6.26 t ha^−1^, while marketable yield ranged from 2.81 to 5.64 t ha^−1^ (Table 3). Here, marketable yield accounted for a much higher percentage of total yield (88%–98%) than for crisphead lettuce. The mean values of marketable and total yield of corn salad in growing period I were 3.19 t ha^−1^ and 3.26 t ha^−1^, in growing period II were 5.15 t ha^−1^ and 5.72 t ha^−1^, and in growing period III were 4.10 t ha^−1^ and 4.26 t ha^−1^. Among the corn salad varieties, regardless of the growing period, the highest marketable yield was in the variety “D'Olanda a seme grosso” (4.7 t ha^−1^), followed by “Ljubljanski” (4.4 t ha^−1^), “Gala” (4.0 t ha^−1^), “Žličar” (3.9 t ha^−1^), and “Pomladin” (3.7 t ha^−1^), but the differences were not significant (Table 4). Analysis of the individual growing periods showed that there was no significant difference between the varieties in marketable yield and total yield in the two spring growing periods, while “Pomladin” and “Gala” had lower marketable yields and total yields than the other three varieties in the autumn open‐field trial (III). Significant differences among the three growing periods (I, II, and III) were found for total yield, waste, and marketable yield of corn salad. The highest marketable yield of corn salad was obtained in spring in the open (II; 5.15 t ha^−1^), followed by autumn in the open (III; 4.10 t ha^−1^) and significantly lower in the spring greenhouse (I; 3.19 t ha^−1^).

The yields achieved are at the level of our earlier trials with crisphead lettuce and corn salad. In the series of trials conducted as part of an expert task Testing of Varieties for the Descriptive List of Varieties with crisphead lettuce from 2004 to 2009 in different growing periods and different Slovenian regions, the spring growing period proved to be the most productive with the lowest percentage of yield losses. Here, average marketable yields ranged from 38 to 75 t ha^−1^ and the percentage of waste yields ranged from 22% to 31%. The trials with local Slovenian crisphead lettuce varieties have also shown that spring is the most productive growing period for these varieties, when some of them can compete with the yields of modern varieties of the same type, as their marketable yields reach up to 40 t ha^−1^. The growth cycle of corn salad was shorter than that of lettuce in both spring growing periods. It took about the same time for corn lettuce to grow from seed to harvest as it did for crisphead lettuce from transplanting to harvest. For both salad crops, the spring growth cycle was shorter in the greenhouse than in the field. Lettuce was harvested 2 weeks earlier in growing period I than in growing period II, while corn salad was harvested about 3 weeks earlier in growing period I than in growing period II. In the experiment with corn salad of the variety “Ljubljanski” in thin‐layer soilless systems in early spring in the greenhouse, harvesting was 47 days after sowing, which is identical to our spring experiment in the greenhouse (Žnidarčič, 2016). It is well known that growing conditions contribute to differences in yield and nutritional quality of lettuce (Rader & Karlsson, 2006).

Color is a very important characteristic for consumers and plays a crucial role in the selection, preference, and acceptance of fresh salad crops. Color parameters (L*, a*, b*, C*, and *hue*) and chlorophyll–SPAD values differed significantly among the salad crop species, varieties, and growing periods (Tables 3). Differences were observed between varieties for all parameters, while between growing periods the only difference observed for crisphead lettuce was for the parameter a* between growing periods I and II (Table 4). The differences between varieties in the parameter *hue* are in good agreement with the visual evaluation of the color of the varieties of both species. In the official descriptions of CPVO (Community Plant Variety Office) and UPOV (International Union for the Protection of New Varieties of Plants) for the lettuce varieties, the green color of the variety “Posavka” is described as dark and for the varieties “Ljubljanska ledenka,” “Leda,” and “Trnovksa ledenka” as light. Among the corn salad varieties, the color of variety “Pomladin” is described as very light (to yellowish) green for “Gala” as medium‐to‐dark green, “Žličar” as medium green, and “D'Olanda a seme grosso” and “Ljubljanski” as light‐to‐medium green. Relative chlorophyll content (chlorophyll–SPAD value) was strongly dependent on variety and growing period. Among the crisphead lettuce varieties, the highest mean relative chlorophyll content was found in “Posavka” (32.70) and the lowest in “Ljubljanska ledenka” (16.82), which means that this difference was almost twofold. As shown in Table 4, the relative chlorophyll content of “Posavka” differed from that of the other crisphead lettuce varieties and from that of growing periods I and II. In the leaves of corn salad, the relative chlorophyll content was higher on average than in those of crisphead lettuce. Among the corn salad varieties, the highest relative content was observed in “Gala,” followed by “D'Olanda a seme grosso,” “Ljubljanski,” “Žličar,” and “Pomladin.” Here, the difference between the highest and the lowest relative chlorophyll content was as in crisphead lettuce, almost twofold. The same crisphead lettuce and corn salad varieties grown in the open field had higher relative chlorophyll contents than those grown in the greenhouse (Table 4). Relative chlorophyll content was highly correlated with most of the color parameters, except for a*. The relative chlorophyll content showed very strong negative correlations with L* (−0.97), b* (−0.85), and C* (−0.81) and a strong negative correlation with *hue* (−0.79) (Table 3).

### 3.2 Nutritional quality

Analysis of total phenolic content (TPC), total sugars, isovaleric acid content, and multielemental composition (macro‐ and microelements) was performed to evaluate nutritional properties. The contents of TPC, total sugars, and isovaleric acid of the studied crisphead lettuce and corn salad varieties grown in different growing periods are summarized in Table 5. TPCs in crisphead lettuce samples varied from 8.9 to 27.3 mg GAE g^−1^ and in corn salad from 22.9 to 35.2 mg GAE g^−1^. Among the crisphead lettuce varieties, regardless of the growing period and leaf position, the highest mean TPC value was in “Ljubljanska ledenka” (15.6 mg GAE g^−1^) and the lowest in “Trnovska ledenka” (12.2 mg GAE g^−1^). Between the different growing periods, TPC in spring crisphead lettuce leaves was higher in the field (II; 15.3 mg GAE g^−1^) than in the greenhouse (I; 12.6 mg GAE g^−1^). TPC in crisphead lettuce decreased with leaf position in the following order: outer > middle > inner (Table 5). Differences between inner and middle leaves were smaller (up to 16%) than for outer leaves (80% and 56%, respectively). Therefore, from a nutritional perspective, consumption of the outer lettuce leaves may significantly increase the intake of total phenolic compounds. Among the corn salad varieties, regardless of the growing period, the highest mean TPC value was determined for “Pomladin” (30.9 mg GAE g^−1^), followed by “D'Olanda a seme grosso” (30.8 mg GAE g^−1^), “Ljubljanski” (30.2 mg GAE g^−1^), “Žličar” (28.3 mg GAE g^−1^), and “Gala” (27.8 mg GAE g^−1^). Significant differences were observed in corn salad between the three growing periods (I, II, and III). The highest TPC between growing periods, regardless of variety, was found for spring greenhouse (I; 33.6 mg GAE g^−1^), followed by spring open field (II; 29.8 mg GAE g^−1^) and autumn open field (III; 25.4 mg GAE g^−1^). Corn salad samples had significantly higher TPCs compared to crisphead lettuce, which is consistent with previous studies on the antioxidant activity assays on this salad crop species (Bunning et al., 2010; Długosz‐Grochowska et al., 2017; Kim et al., 2018; Liu et al., 2007).

**TABLE 5:** Total phenolic content, total sugars, and isovaleric acid of four crisphead lettuce varieties cultivated in two growing periods (depending on leaf position) and five corn salad varieties cultivated in three growing periods

Variety	Growing period	Leaf position	TPC (mg GAE g^−1^)	Total sugars (g kg^−1^)	Valerenic acid (g kg^−1^)
Crisphead lettuce					
Posavka	I	Outer	15.7	138.2	n.d.
		Middle	12.3	258.2	n.d.
		Inner	11.4	346.0	n.d.
	II	Outer	17.8	171.8	n.d.
		Middle	13.1	319.0	n.d.
		Inner	11.0	342.0	n.d.
	*Mean*		*13.6*	*262.5*	*n.d*.
Ljubljanska ledenka	I	Outer	21.7	194.9	n.d.
		Middle	10.6	281.3	n.d.
		Inner	10.0	360.0	n.d.
	II	Outer	27.3	145.0	n.d.
		Middle	13.1	312.0	n.d.
		Inner	11.1	384.0	n.d.
	*Mean*		*15.6*	*279.5*	*n.d*.
Leda	I	Outer	17.6	102.7	n.d.
		Middle	11.7	317.0	n.d.
		Inner	9.9	318.0	n.d.
	II	Outer	19.5	147.8	n.d.
		Middle	14.9	363.0	n.d.
		Inner	12.1	377.0	n.d.
	*Mean*		*14.3*	*270.9*	*n.d*.
Trnovska ledenka	I	Outer	11.4	158.4	n.d.
		Middle	9.5	286.1	n.d.
		Inner	8.9	367.0	n.d.
	II	Outer	21.1	134.4	n.d.
		Middle	12.4	333.0	n.d.
		Inner	9.9	476.0	n.d.
	*Mean*		*12.2*	*292.5*	*n.d*.
*Mean I*			*12.6*	*260.7*	*n.d*.
*Mean II*			*15.3*	*292.1*	*n.d*.
Corn salad					
Ljubljanski	I	n.a.	34.7	44.2	8.5
	II	n.a.	32.4	88.0	7.1
	III	n.a.	23.4	192.0	2.6
	*Mean*		*30.2*	*108.1*	*6.1*
Žličar	I	n.a.	33.1	30.0	8.1
	II	n.a.	26.3	107.0	5.9
	III	n.a.	25.6	192.0	3.6
	*Mean*		*28.3*	*109.7*	*5.9*
Pomladin	I	n.a.	33.0	50.1	5.0
	II	n.a.	32.1	54.7	3.5
	III	n.a.	27.6	138.2	2.9
	*Mean*		*30.9*	*81.0*	*3.8*
Gala	I	n.a.	32.1	55.3	9.0
	II	n.a.	28.5	85.6	7.5
	III	n.a.	22.9	161.3	3.8
	*Mean*		*27.8*	*100.7*	*6.8*
D'Olanda a seme grosso	I	n.a.	35.2	53.4	7.1
	II	n.a.	29.8	100.8	6.9
	III	n.a.	27.5	162.2	4.8
	*Mean*		*30.8*	*105.5*	*6.3*
*Mean I*		*n.a*.	*33.6*	*46.6*	*7.5*
*Mean II*		*n.a*.	*29.8*	*87.2*	*6.2*
*Mean III*		*n.a*.	*25.4*	*169.1*	*3.5*

*Note*: Data are mean (*n* = 3).

Abbreviations: I, spring (greenhouse); II, spring (open field); III, autumn (open field); GAE, gallic acid equivalents; n.d., not detected; TPC, total phenolic content.

The total sugar concentration in the crisphead lettuce samples varied considerably from 102.7 to 476.0 g kg^−1^ and in corn salad from 30.0 to 192.0 g kg^−1^, with the mean total sugar contents of the crisphead lettuce samples being approximately 2.7 times higher compared to the corn salad samples. Among the crisphead lettuce varieties, regardless of the growing period and leaf position, the mean total sugar content was highest in “Trnovska ledenka” (292.5 g kg^−1^) and lowest in “Posavka” (262.5 g kg^−1^). Between the different growing periods, the total sugars in leaves of crisphead lettuce grown in spring in the open (II; 292.1 g kg^−1^) were slightly higher than in spring grown in the greenhouse (I; 260.7 g kg^−1^). Total sugars in crisphead lettuce decreased with leaf position in the following order: inner > middle > outer (Table 5). Differences between inner and middle leaves were smaller (up to 20%) than for outer leaves (149% and 107%, respectively). Among the corn salad varieties, regardless of the growing period, the highest mean total sugar content was found for “Žličar” (109.7 g kg^−1^) and the lowest for “Pomladin” (81.0 g kg^−1^). Significant differences were found in corn salad among three growing periods (I, II, and III). The highest mean TPC between growing periods, regardless of variety, was found for the autumn open field (III; 169.1 g kg^−1^), followed by spring open field (II; 87.2 g kg^−1^) and spring greenhouse (I; 46.6 g kg^−1^).

There are no data yet on (iso)valeric acid, which is responsible for the characteristic aroma (Patočka & Jakl, 2010), in corn salad varieties produced in different growing periods. Although valeric acid from the genus *Valeriana officinalis* has demonstrated anxiolytic activity, there is limited information on valeric acid from the genus *V. locusta* (Becker et al., 2014; Felgentreff et al., 2012). Isovaleric acid and valeric acid were measured in crisphead lettuce and corn salad samples; however, isovaleric acid was present only in corn salad, whereas it was not detected in crisphead lettuce. Isovaleric acid content in corn salad samples ranged from 2.6 to 9.0 g kg^−1^. The highest mean isovaleric acid content between growing periods, regardless of variety, was found in the spring greenhouse (I; 7.5 g kg^−1^), followed by spring open field (II; 6.2 g kg^−1^) and autumn open field (III; 3.5 g kg^−1^).

The multielement composition of crisphead lettuce and corn salad varieties determined using ICP‐MS is shown in Table 6. A total of 10 elements were determined and divided into five macroelements (Mg, P, S, K, and Ca) and five microelements (Cr, Mn, Fe, Zn, and Mo). The results of macroelements are given as g kg^−1^ and those of microelements as mg kg−1 (Table 6). Based on the mean values, the order of elements is K > Ca > P > Mg > S > Fe > Zn > Mn > Mo > Cr in the studied salad crops samples. The ranking of individual macroelements in the analyzed leaves of crisphead lettuce and corn salad was as follows: K (32.2–79.9 g kg^−1^), Ca (2.8–15.0 g kg^−1^), P (4.4–7.1 g kg^−1^), Mg (1.1–6.0 g kg^−1^), and S (1.0–3.5 g kg^−1^). The greatest differences among salad crop species were for S, which was higher to some extent in corn salad leaves. The ranking of microelements in the analyzed salad crops was as follows: Fe (46.3–402.7 mg kg^−1^), Zn (16.8–55.5 mg kg^−1^), Mn (10.1–46.9 mg kg^−1^), Mo (0.3–4.0 mg kg^−1^), and Cr (0.2–4.4 mg kg^−1^). Of the microelements determined, the greatest differences between the two salad crops were found for Fe, which was higher in the leaves of corn salad. These elemental compositions are consistent with literature data for crisphead lettuce (Kim et al., 2016) and corn salad leaves (Gottardi et al., 2012). The results showed that the outer leaves of crisphead lettuce contained significantly higher concentrations of macro‐ and microelements, except for P and Fe (Table 4). The growing period affected both macro‐ and microelements in the leaves of corn salad. The concentrations of the elements Mg, S, K, Ca, and Zn were significantly higher in the autumn open‐field growing period.

**TABLE 6:** Multielement composition of four crisphead lettuce varieties cultivated in two growing periods (depending on leaf position) and five corn salad varieties cultivated in three growing periods

Variety	Growing period	Leaf position	Macroelement (g kg^−1^)					Microelement (mg kg^−1^)				
			Mg	P	S	K	Ca	Cr	Mn	Fe	Zn	Mo
Crisphead lettuce												
Posavka	I	Outer	5.5	4.6	1.8	73.6	15.0	0.8	35.7	82.3	18.3	0.9
		Middle	2.8	7.1	2.1	47.9	6.6	0.2	25.1	79.2	41.6	0.6
		Inner	2.1	6.1	1.6	36.7	5.0	0.5	18.7	65.9	36.6	0.5
	II	Outer	3.3	4.7	1.7	65.6	13.2	0.7	22.4	163.0	27.0	1.8
		Middle	1.4	5.8	1.3	36.3	3.7	0.5	12.1	79.5	34.8	0.6
		Inner	1.6	6.2	1.6	36.9	3.3	0.3	13.1	72.1	41.2	0.6
	*Mean*		*2.8*	*5.8*	*1.7*	*49.5*	*7.8*	*0.5*	*21.2*	*90.3*	*33.3*	*0.8*
Ljubljanska ledenka	I	Outer	3.1	5.0	1.7	67.7	10.6	0.3	25.6	82.8	26.9	0.8
		Middle	2.1	6.3	1.6	51.6	5.7	0.3	19.0	73.0	35.2	0.5
		Inner	1.7	5.7	1.6	46.6	4.3	0.2	14.9	50.9	33.9	0.4
	II	Outer	2.5	5.9	2.0	79.9	11.6	0.5	21.1	103.4	35.7	2.1
		Middle	1.4	5.9	1.0	44.7	4.0	0.3	13.6	62.3	29.9	0.6
		Inner	1.2	5.5	1.0	35.4	3.0	0.2	10.1	46.5	33.9	0.6
	*Mean*		*2.0*	*5.7*	*1.5*	*54.3*	*6.5*	*0.3*	*17.4*	*69.8*	*32.6*	*0.8*
Leda	I	Outer	3.6	5.6	2.0	74.7	11.4	0.7	28.8	91.4	29.6	1.6
		Middle	1.8	6.7	1.2	48.3	4.1	0.4	17.6	74.6	41.7	0.7
		Inner	1.8	6.3	1.4	43.7	3.9	0.4	17.2	64.5	39.1	0.9
	II	Outer	2.9	4.7	1.8	78.0	8.7	0.6	22.2	165.0	16.8	0.8
		Middle	1.6	5.5	1.2	46.5	5.6	0.6	15.6	98.3	21.9	0.7
		Inner	1.4	5.3	1.5	38.5	3.1	0.2	11.5	59.6	29.5	0.3
	*Mean*		*2.2*	*5.7*	*1.5*	*54.9*	*6.1*	*0.5*	*18.8*	*92.2*	*29.8*	*0.8*
Trnovska ledenka	I	Outer	4.1	4.4	1.8	78.0	11.8	0.7	30.9	96.1	55.5	1.4
		Middle	2.4	5.7	1.1	51.9	5.8	0.3	20.7	82.4	26.3	0.7
		Inner	1.9	5.7	1.3	43.0	4.7	0.3	17.7	71.8	30.3	0.7
	II	Outer	2.6	5.3	2.4	74.4	11.5	0.7	22.5	231.6	17.1	1.4
		Middle	1.5	5.8	1.0	47.9	3.7	0.2	13.3	59.8	26.4	0.4
		Inner	1.1	5.1	1.1	32.2	2.8	0.2	10.2	46.3	29.0	0.4
	*Mean*		*2.3*	*5.3*	*1.4*	*54.6*	*6.7*	*0.4*	*19.2*	*98.0*	*30.8*	*0.8*
*Mean I*			*2.7*	*5.8*	*1.6*	*55.3*	*7.4*	*0.4*	*22.7*	*76.2*	*34.6*	*0.8*
*Mean II*			*1.9*	*5.5*	*1.5*	*51.4*	*6.2*	*0.4*	*15.6*	*99.0*	*28.6*	*0.9*
Corn salad												
Ljubljanski	I	n.a.	4.7	5.0	2.9	59.9	10.4	0.9	38.9	186.2	41.9	0.9
	II	n.a.	3.5	4.9	2.8	50.8	8.6	0.8	35.7	297.2	25.2	2.2
	III	n.a.	3.9	5.7	1.9	34.9	5.9	1.2	32.9	292.0	32.7	3.8
	*Mean*		*4.0*	*5.2*	*2.5*	*48.5*	*8.3*	*1.0*	*35.8*	*258.5*	*33.3*	*2.3*
Žličar	I	n.a.	5.0	5.3	3.5	55.7	10.2	0.5	33.5	166.7	47.4	1.3
	II	n.a.	3.4	4.7	2.6	45.6	8.6	0.9	27.5	241.2	26.4	2.4
	III	n.a.	4.2	5.4	1.9	35.0	5.9	1.0	29.6	246.8	35.5	3.7
	*Mean*		*4.2*	*5.1*	*2.7*	*45.4*	*8.2*	*0.8*	*30.2*	*218.2*	*36.4*	*2.5*
Pomladin	I	n.a.	4.5	6.0	2.8	69.0	13.9	0.7	30.0	272.1	41.7	0.9
	II	n.a.	3.9	4.4	2.7	57.4	14.4	0.8	46.9	343.5	22.7	2.9
	III	n.a.	3.0	6.0	2.3	40.9	7.8	3.7	24.1	402.7	27.9	2.2
	*Mean*		*3.8*	*5.5*	*2.6*	*55.8*	*12.0*	*1.7*	*33.7*	*339.4*	*30.8*	*2.0*
Gala	I	n.a.	6.0	5.4	3.0	56.1	12.5	0.7	39.7	208.7	46.7	1.4
	II	n.a.	4.0	5.1	2.6	47.2	9.7	1.3	28.2	278.6	24.2	2.8
	III	n.a.	5.0	5.7	1.8	34.6	7.4	4.4	24.0	253.1	35.2	4.0
	*Mean*		*5.0*	*5.4*	*2.4*	*45.9*	*9.9*	*2.1*	*30.6*	*246.8*	*35.4*	*2.7*
D'Olanda a seme grosso	I	n.a.	5.1	5.0	2.7	57.4	10.6	0.6	35.5	248.3	54.3	1.5
	II	n.a.	4.0	5.3	2.7	52.3	8.9	0.8	27.5	256.9	24.9	3.3
	III	n.a.	4.9	5.5	1.9	35.5	7.9	1.2	25.3	201.0	31.2	3.9
	*Mean*		*4.7*	*5.3*	*2.5*	*48.4*	*9.1*	*0.8*	*29.4*	*235.4*	*36.8*	*2.9*
*Mean I*			*5.1*	*5.3*	*3.0*	*59.6*	*11.5*	*0.7*	*35.5*	*216.4*	*46.4*	*1.2*
*Mean II*			*3.7*	*4.9*	*2.7*	*50.6*	*10.0*	*0.9*	*33.2*	*283.5*	*24.7*	*2.7*
*Mean III*			*4.2*	*5.7*	*2.0*	*36.1*	*7.0*	*2.3*	*27.2*	*279.1*	*32.5*	*3.5*

Abbreviations: I, spring (greenhouse); II, spring (open field); III, autumn (open field); n.a., not applicable.

## 4 CONCLUSION

There is a need for reliable nutritional data on important salad crops such as crisphead lettuce and corn salad to provide information on nutrient uptake. In addition, agronomic parameters (yield and color) provide important information for growers and can potentially be used as a basis for deciding whether to grow a particular variety. This study provides interesting comparisons between different growing periods and leaf positions of crisphead lettuce and corn salad varieties grown in the soil. The growing period affected yield for all varieties studied only for corn salad, with the highest yield determined in the open field in spring. Interestingly, total sugar contents were highest in the samples with the lowest TPC, and vice versa, in both salad crop species. The most significant differences were observed in the leaf position parameter. Overall, the outer crisphead leaves had higher nutritional quality than the inner leaves. This study provides complex nutritional data for *L. sativa* and *Valerianella* spp. cultivated in soil during several growing periods, as well as relationships between species.

## CONFLICT OF INTEREST

The authors have declared no conflict of interest.

## DATA AVAILABILITY STATEMENT.

The data supporting the results of this study were provided in the form of tables and figures. All authors state that additional data will be made available upon request to the corresponding author.

## ETHICAL APPROVAL

This study does not involve any human or animal testing.

## References

[fsn33205-bib-0001] Becker, A. , Felgentreff, F. , Schröder, H. , Meier, B. , & Brattström, A. (2014). The anxiolytic effects of a valerian extract is based on Valerenic acid. BMC Complementary and Alternative Medicine, 14(1), 1–5. 10.1186/1472-6882-14-267 25066015 PMC4122768

[fsn33205-bib-0002] Bunning, M. L. , Kendall, P. A. , Stone, M. B. , Stonaker, F. H. , & Stushnoff, C. (2010). Effects of seasonal variation on sensory properties and total phenolic content of 5 lettuce cultivars. Journal of Food Science, 75(3), 156–161. 10.1111/j.1750-3841.2010.01533.x 20492312

[fsn33205-bib-0003] Camejo, D. , Frutos, A. , Mestre, T. C. , del Carmen Piñero, M. , Rivero, R. M. , & Martínez, V. (2020). Artificial light impacts the physical and nutritional quality of lettuce plants. Horticulture, Environment, and Biotechnology, 61(1), 69–82. 10.1007/s13580-019-00191-z

[fsn33205-bib-0004] Ceglie, F. G. , Amodio, M. L. , de Chiara, M. L. V. , Madzaric, S. , Mimiola, G. , Testani, E. , Tittarelli, F. , & Colelli, G. (2018). Effect of organic agronomic techniques and packaging on the quality of lamb's lettuce. Journal of the Science of Food and Agriculture, 98(12), 4606–4615. 10.1002/jsfa.8989 29508398

[fsn33205-bib-0005] Colonna, E. , Rouphael, Y. , Barbieri, G. , & De Pascale, S. (2016). Nutritional quality of ten leafy vegetables harvested at two light intensities. Food Chemistry, 199, 702–710. 10.1016/j.foodchem.2015.12.068 26776027

[fsn33205-bib-0006] Długosz‐Grochowska, O. , Wojciechowska, R. , Kruczek, M. , & Habela, A. (2017). Supplemental lighting with LEDs improves the biochemical composition of two *Valerianella locusta* (L.) cultivars. Horticulture, Environment, and Biotechnology, 58(5), 441–449. 10.1007/s13580-017-0300-4

[fsn33205-bib-0007] EC No 152/2009 Commission regulation laying down the methods of sampling and analysis for the official control of feed. Official Journal of the European Union L54/1:1–130 http://eurlex.europa.eu/LexUriServ/LexUriServ.do?uri=OJ:L:2009:054:0001:0130:EN:PDF

[fsn33205-bib-0008] Felgentreff, F. , Becker, A. , Meier, B. , & Brattström, A. (2012). Valerian extract characterized by high valerenic acid and low acetoxy valerenic acid contents demonstrates anxiolytic activity. Phytomedicine, 19(13), 1216–1222. 10.1016/j.phymed.2012.08.003 22944521

[fsn33205-bib-0009] García‐Valcárcel, A. I. , Loureiro, I. , Escorial, C. , Molero, E. , & Tadeo, J. L. (2016). Uptake of azoles by lamb's lettuce (*Valerianella locusta* L.) grown in hydroponic conditions. Ecotoxicology and Environmental Safety, 124, 138–146. 10.1016/j.ecoenv.2015.10.021 26513529

[fsn33205-bib-0010] Gottardi, S. , Iacuzzo, F. , Tomasi, N. , Cortella, G. , Manzocco, L. , Pinton, R. , Römheld, V. , Mimmo, T. , Scampicchio, M. , Dalla Costa, L. , & Cesco, S. (2012). Beneficial effects of silicon on hydroponically grown corn salad (*Valerianella locusta* (L.) Laterr) plants. Plant Physiology and Biochemistry, 56, 14–23. 10.1016/j.plaphy.2012.04.002 22579940

[fsn33205-bib-0011] Hernández, V. , Botella, M. Á. , Hellín, P. , Cava, J. , Fenoll, J. , Mestre, T. , Martínez, V. , & Flores, P. (2021). Phenolic and carotenoid profile of lamb's lettuce and improvement of the bioactive content by preharvest conditions. Food, 10(1), 188. 10.3390/foods10010188 PMC7831921 33477681

[fsn33205-bib-0012] Hoy, M. K. , Sebastian, R. S. , Goldman, J. D. , Wilkinson Enns, C. , & Moshfegh, A. J. (2019). Consuming vegetable‐based salad is associated with higher nutrient intakes and diet quality among US adults, what we eat in America, National Health and nutrition examination survey 2011‐2014. Journal of the Academy of Nutrition and Dietetics, 119(12), 2085–2092. 10.1016/j.jand.2019.04.018 31278048

[fsn33205-bib-0013] Jenni, S. , Truco, M. J. , & Michelmore, R. W. (2013). Quantitative trait loci associated with tipburn, heat stress‐induced physiological disorders, and maturity traits in crisphead lettuce. Theoretical and Applied Genetics, 126, 3065–3079. 10.1007/s00122-013-2193-7 24078012

[fsn33205-bib-0014] Kang, Y. R. , Park, J. , Jung, S. K. , & Chang, Y. H. (2018). Synthesis, characterization, and functional properties of chlorophylls, pheophytins, and Zn‐pheophytins. Food Chemistry, 245, 943–950. 10.1016/j.foodchem.2017.11.079 29287463

[fsn33205-bib-0015] Kim, D. E. , Shang, X. , Assefa, A. D. , Keum, Y. S. , & Saini, R. K. (2018). Metabolite profiling of green, green/red, and red lettuce cultivars: Variation in health beneficial compounds and antioxidant potential. Food Research International, 105, 361–370. 10.1016/j.foodres.2017.11.028 29433225

[fsn33205-bib-0016] Kim, M. J. , Moon, Y. , Tou, J. C. , Mou, B. , & Waterland, N. L. (2016). Nutritional value, bioactive compounds and health benefits of lettuce (*Lactuca sativa* L.). Journal of Food Composition and Analysis, 49, 19–34. 10.1016/j.jfca.2016.03.004

[fsn33205-bib-0017] Kotsiras, A. , Vlachodimitropoulou, A. , Gerakaris, A. , Bakas, N. , & Darras, A. I. (2016). Innovative harvest practices of Butterhead, Lollo Rosso and Batavia green lettuce (*Lactuca sativa* L.) types grown in floating hydroponic system to maintain the quality and improve storability. Scientia Horticulturae, 201, 1–9. 10.1016/j.scienta.2016.01.021

[fsn33205-bib-0018] Křístková, E. , Doležalová, I. , Lebeda, A. , Vinter, V. , & Novotna, A. (2008). Description of morphological characters of lettuce (*Lactuca sativa* L.) genetic resources. Horticultural Science (Prague), 35(3), 113–129.

[fsn33205-bib-0019] Lanje, C. N. , Patil, S. R. , & Wankhade, A. M. (2020). Medicinal natural drug of valerian (*Valerina officinalis*): An‐over review. American Journal of PharmTech Research, 10(1), 148–173. 10.46624/ajptr.2020.v10.i1.013

[fsn33205-bib-0020] Lee, S. M. , Lee, K. T. , Lee, S. H. , & Song, J. K. (2013). Origin of human colour preference for food. Journal of Food Engineering, 119(3), 508–515. 10.1016/j.jfoodeng.2013.06.021

[fsn33205-bib-0021] Liu, X. , Ardo, S. , Bunning, M. , Parry, J. , Zhou, K. , Stushnoff, C. , Stoniker, F. , Yu, L. , & Kendall, P. (2007). Total phenolic content and DPPH˙ radical scavenging activity of lettuce (*Lactuca sativa* L.) grown in Colorado. LWT ‐ Food Science and Technology, 40(3), 552–557. 10.1016/j.lwt.2005.09.007

[fsn33205-bib-0022] Markovic, I. , Ilic, J. , Markovic, D. , Simonovic, V. , & Kosanic, N. (2013). Color measurment of food products using CIE *L***a***b** and RGB color space. Journal of Hygienic Engineering and Design, 4(1), 50–53.

[fsn33205-bib-0023] Masarirambi, M. T. , Nxumalo, K. A. , Musi, P. J. , & Rugube, L. M. (2018). Common physiological disorders of lettuce (*Lactuca sativa*) found in Swaziland: A review. American‐Eurasian Journal of Agricultural and Environmental Sciences, 18(1), 50–56. 10.5829/idosi.aejaes.2018.50.56

[fsn33205-bib-0024] Mou, B. (2008). Lettuce. In J. Prohens & F. Nuez (Eds.), Handbook of plant breeding, Vol. I vegetables I: Asteraceae, ‐Brassicaceae, Chenopodicaceae, and Cucurbitaceae (pp. 75–115). Springer https://scholar.google.com/scholar?hl=en&as_sdt=0%2C5 &q=9.%09Mou%2C+B.%2C+2008.+Lettuce.+In%3A+Prohens%2C+F.+Nuez+%28Eds.%29%2C+Handbook+of+Plant+Breeding%2C+Vol.+I+Vegetables+I%3A+Asteraceae%2C+‐Brassicaceae%2C+Chenopodicaceae%2C+and+Cucurbitaceae.+Sprin

[fsn33205-bib-0025] Mou, B. (2012). Nutritional quality of lettuce. Current Nutrition & Food Science, 8(3), 177–187. 10.2174/157340112802651121

[fsn33205-bib-0026] Negrao, L. D. , Sousa, P. V. L. , Barradas, A. M. , Brandao, A. C. A. S. , Araujo, M. A. M. , & Moreira‐Araujo, R. S. R. (2021). Bioactive compounds and antioxidant activity of crisphead lettuce (*Lactuca sativa* L.) of three different cultivation systems. Food Science and Technology, 41(2), 365–370. 10.1590/fst.04120

[fsn33205-bib-0027] Noumedem, J. A. K. , Djeussi, D. E. , Hritcu, L. , Mihasan, M. , & Kuete, V. (2017). Lactuca sativa. In Medicinal spices and vegetables from Africa (pp. 437–449). Elsevier. 10.1016/B978-0-12-809286-6.00020-0

[fsn33205-bib-0028] Pathare, P. B. , Opara, U. L. , & Al‐Said, F. A. J. (2013). Colour measurement and analysis in fresh and processed foods: A review. Food and Bioprocess Technology, 6(1), 36–60. 10.1007/s11947-012-0867-9

[fsn33205-bib-0029] Patočka, J. , & Jakl, J. (2010). Biomedically relevant chemical constituents of *Valeriana officinalis* . Journal of Applied Biomedicine, 8(1), 11–18. 10.2478/v10136-009-0002-z

[fsn33205-bib-0030] Pinotti, L. , Manoni, M. , Fumagalli, F. , Rovere, N. , Luciano, A. , Ottoboni, M. , Ferrari, L. , Cheli, F. , & Djuragic, O. (2020). Reduce, reuse, recycle for food waste: A second life for fresh‐cut leafy salad crops in animal diets. Animals, 10(6), 1–14. 10.3390/ani10061082 PMC7341183 32585906

[fsn33205-bib-0031] Rader, H. B. , & Karlsson, M. G. (2006). Northern field production of leaf and romaine lettuce using a high tunnel. HortTechnology, 16(4), 649–654. 10.21273/horttech.16.4.0649

[fsn33205-bib-0032] Sahin, U. , Kuslu, Y. , Kiziloglu, F. M. , & Cakmakci, T. (2016). Growth, yield, water use and crop quality responses of lettuce to different irrigation quantities in a semi‐arid region of high altitude. Journal of Applied Horticulture, 18(3), 195–202 www.horticultureresearch.net

[fsn33205-bib-0033] Schvambach, M. I. , Vargas Andriolli, B. , Fernandes de Souza, P. , Barcelos Oliveira, J. L. , & Pescador, R. (2020). Conservation of crisp lettuce in different post‐harvest storage conditions. Revista Ceres, 67(4), 247–250.

[fsn33205-bib-0034] Shi, F. , Li, Y. , Han, R. , Fu, A. , Wang, R. , Nusbaum, O. , Qin, Q. , Chen, X. , Hou, L. , & Zhu, Y. (2021). Valerian and valeric acid inhibit growth of breast cancer cells possibly by mediating epigenetic modifications. Scientific Reports, 11(1), 1–10. 10.1038/s41598-021-81620-x 33510252 PMC7844014

[fsn33205-bib-0035] Singleton, V. L. , & Rossi, J. A. (1965). Colorimetry of total phenolics with phosphomolybdic‐phosphotungstic acid reagents. American Journal of Enology and Viticulture, 16(3), 144–158.

[fsn33205-bib-0036] Sucheta, A. , Singla, G. , Chaturvedi, K. , & Sandhu, P. P. (2019). Status and recent trends in fresh‐cut fruits and vegetables. In Fresh‐cut fruits and vegetables: Technologies and mechanisms for safety control (pp. 17–49). Elsevier Inc.. 10.1016/B978-0-12-816184-5.00002-1

[fsn33205-bib-0037] Šuštar‐Vozlič, J. , Ugrinović, K. , Maras, M. , Křístková, E. , Lebeda, A. , & Meglič, V. (2021). Morphological and genetic diversity of Slovene lettuce landrace ‘Ljubljanska ledenka' (*Lactuca sativa* L.). Genetic Resources and Crop Evolution, 68(1), 185–203. 10.1007/s10722-020-00978-5

[fsn33205-bib-0038] Wei, T. , van Treuren, R. , Liu, X. , Zhang, Z. , Chen, J. , Liu, Y. , Dong, S. , Sun, P. , Yang, T. , Lan, T. , Wang, X. , Xiong, Z. , Liu, Y. , Wei, J. , Lu, H. , Han, S. , Chen, J. C. , Ni, X. , Wang, J. , & Liu, H. (2021). Whole‐genome resequencing of 445 Lactuca accessions reveals the domestication history of cultivated lettuce. Nature Genetics, 53(5), 752–760. 10.1038/s41588-021-00831-0 33846635

[fsn33205-bib-0039] Yang, X. , Feng, L. , Zhao, L. , Liu, X. , Hassani, D. , & Huang, D. (2018). Effect of glycine nitrogen on lettuce growth under soilless culture: A metabolomics approach to identify the main changes occurred in plant primary and secondary metabolism. Journal of the Science of Food and Agriculture, 98(2), 467–477. 10.1002/jsfa.8482 28612342

[fsn33205-bib-0040] Zhou, W. , Liang, X. , Zhang, Y. , Dai, P. , Liang, B. , Li, J. , Sun, C. , & Lin, X. (2020). Role of sucrose in modulating the low‐nitrogen‐induced accumulation of phenolic compounds in lettuce (*Lactuca sativa* L.). Journal of the Science of Food and Agriculture, 100(15), 5412–5421. 10.1002/jsfa.10592 32562270

[fsn33205-bib-0041] Žnidarčič, D. (2016). Evaluation of yield and yield components of lamb's lettuce (*Valerianella locusta*) grown in thin layer soilless systems. Nusantara Bioscience, 8(1), 89–93. 10.13057/nusbiosci/n080116

